# Fast Diffusion Sustains Plasma Membrane Accumulation of Phosphatase of Regenerating Liver-1

**DOI:** 10.3389/fcell.2020.585842

**Published:** 2020-12-04

**Authors:** Patricia Castro-Sánchez, Sara Hernández-Pérez, Oscar Aguilar-Sopeña, Rocia Ramírez-Muñoz, Sandra Rodríguez-Perales, Raúl Torres-Ruiz, Pedro Roda-Navarro

**Affiliations:** ^1^Department of Immunology, Ophthalmology and ENT, School of Medicine, Universidad Complutense de Madrid and 12 de Octubre Health Research Institute (imas12), Madrid, Spain; ^2^Molecular Cytogenetics and Genome Editing Unit, Human Cancer Genetics Program, Centro Nacional de Investigaciones Oncológicas (CNIO), Madrid, Spain

**Keywords:** phosphatases of regenerating liver, farnesylated protein, fluorescence recovery after photobleaching, fluorescence correlation spectroscopy, CRISPR/Cas9, genome edition

## Abstract

It has been proposed that the accumulation of farnesylated phosphatase of regenerating liver-1 (PRL-1) at the plasma membrane is mediated by static electrostatic interactions of a polybasic region with acidic membrane lipids and assisted by oligomerization. Nonetheless, localization at early and recycling endosomes suggests that the recycling compartment might also contribute to its plasma membrane accumulation. Here, we investigated in live cells the dynamics of PRL-1 fused to the green fluorescent protein (GFP-PRL-1). Blocking the secretory pathway and photobleaching techniques suggested that plasma membrane accumulation of PRL-1 was not sustained by recycling endosomes but by a dynamic exchange of diffusible protein pools. Consistent with this idea, fluorescence correlation spectroscopy in cells overexpressing wild type or monomeric mutants of GFP-PRL-1 measured cytosolic and membrane-diffusing pools of protein that were not dependent on oligomerization. Endogenous expression of GFP-PRL-1 by CRISPR/Cas9 genome edition confirmed the existence of fast diffusing cytosolic and membrane pools of protein. We propose that plasma membrane PRL-1 replenishment is independent of the recycling compartment and the oligomerization state and mainly driven by fast diffusion of the cytosolic pool.

## Introduction

Phosphatases of regenerating liver (PRLs), PRL-1, PRL-2, and PRL-3, constitute a group of three dual specificity phosphatases encoded by the genes *PTP4A1*, *PTP4A2*, and *PTP4A3*, respectively, and whose high expression is associated to cancer progression (Hardy et al., 2018). PRLs contain a polybasic region and a CAAX motif for farnesylation that allows targeting to cell membranes in the endoplasmic reticulum (ER), the endosomal compartment and the plasma membrane, where PRLs are typically enriched (Zeng et al., 2000; Wang et al., 2002). Mutations of the CAAX motifs redirect PRLs to the nucleus, suggesting that the polybasic region also targets the protein to this compartment (Zeng et al., 2000; Wang et al., 2002). Neither the polybasic region nor the CAAX motif alone are sufficient for cell membrane localisation, as mutants lacking one of these motifs, as well as the treatment with farnesyltransferase inhibitors, renders soluble expression of PRLs (Zeng et al., 2000; Wang et al., 2002; Sun et al., 2005; Rios et al., 2013; Castro-Sanchez et al., 2018).

CAAX motif-containing proteins are farnesylated by cytosolic farnesyltransferases and this post-translational modification promotes the binding to the ER membranes (Wang and Casey, 2016). ER membranes are much more abundant than the plasma membrane and compete for the binding of hydrophobic farnesylated PRLs. Therefore, it is plausible the existence of an active mechanism explaining the plasma membrane enrichment of these phosphatases. Although it is mainly thought that the plasma membrane enrichment of PRLs is mediated by a static membrane binding provided by electrostatic interactions of the polybasic region and by the protein insertion in the bilayer by farnesylation, the mechanism involved has not been deeply investigated. Several mechanisms might be involved, including traffic in the conventional secretory pathway and the endosomal compartment, conformational changes and oligomerization, post-translational modifications and direct diffusion-mediated transport.

Crystallization of PRL-1 has revealed the formation of trimmers (Jeong et al., 2005). Although trimerization occurs in membrane fractions, it is not known whether this configuration has a regulatory role on its phosphatase activity against natural substrates in the cell. Farnesylation is necessary for trimerization, probably by raising local protein concentration at cell membranes (Jeong et al., 2005). Trimerization might contribute to a stronger stabilization of PRLs at cell membranes by adding more hydrophobic forces on the monomer and, for this reason, it has been proposed by some authors to regulate the traffic of the protein to the plasma membrane (Jeong et al., 2005). By contrast, other authors find trimerization not necessary for the presence of PRL-1 in membrane fractions of cell extracts (Sun et al., 2005). Thus, this question deserves further investigation.

In order to test potential mechanisms for PRL-1 plasma membrane enrichment, we studied the localisation and dynamics of GFP-PRL-1 fluorescent fusion proteins in live cells. Initially, due to the distribution of PRL-1 at the plasma membrane, early endosomes (Zeng et al., 2000) and recycling endosomes in T cells (Castro-Sanchez et al., 2018), we aimed to study the contribution of the recycling compartment. Secondly, we evaluated the dynamics of GFP-PRL-1 at the plasma membrane by photobleaching methods. Then, we explored the existence of diffusible pools of GFP-PRL-1 by fluorescence correlation spectroscopy (FCS). In order to compare the diffusion properties of GFP-PRL-1 trimers and monomers, we used mutants of critical residues of the dimerization interface rendering the protein unable to form trimmers (Jeong et al., 2005; Sun et al., 2005). Finally, to investigate potential alterations of GFP-PRL-1 dynamics due to protein overexpression, we edited the *PTP4A1* gene to generate an open reading frame coding for the GFP-PRL-1 fluorescent fusion protein under endogenous levels. The obtained results suggest a diffusion-mediated rapid transport of PRL-1 from the cytosol to the plasma membrane independent of the recycling compartment and the oligomerization state.

## Materials and Methods

### Cell Culture, Antibodies and Reagents

The Jurkat CD4 T cell line (JK) and the Raji B cell line (American Type Culture Collection) were grown at 37°C and 5% CO2 in RPMI 1640 medium (Lonza Group, Switzerland), supplemented with 10% inactivated fetal calf serum (FCS) (Gibco, Gaithersburg, MD, United States), 10 mM glutamine, 100 U/mL penicillin and 100 μg/ml streptomycin (all from Lonza Group). Mouse anti-CD3 monoclonal antibody was provided by Dr. Francisco Sánchez-Madrid (Hospital Universitario de la Princesa, Madrid, Spain); mouse anti-PRL-1/2 (clone 42) monoclonal antibody was purchased from Millipore (Burlington, MA, United States); mouse anti-CD71 was from BD Bioscience (San Jose, CA, United States) and rabbit anti-GFP polyclonal antibody was purchased from Life Technologies (Carlsbad, CA, United States). Secondary antibody goat anti-mouse-Ig Alexa Fluor 594 (highly cross-absorbed) and the fluorescent tracker 7-amino-4-chloromethyl coumarin (CMAC) were obtained from Molecular probes (Eugene, OR, United States). Poly-L-lysine, Mowiol and brefeldin A (BFA) were obtained from Sigma Aldrich (St. Louis, MO, United States) and *Staphylococcus* Enterotoxin E (SEE) from Toxin Technologies (Sarasota, FL, United States).

### Expression Vectors, Transfection and Genome Edition

The GFP-PRL-1, GFP-PRL-1_ΔCAAX, and 2B4-GFP expressing plasmids were previously developed in our laboratories using the plasmid pEGFP-C1 (PRL-1 plasmids) and pEGFP-N1 (2B4 plasmid) (Clontech) (Roda-Navarro et al., 2004; Castro-Sanchez et al., 2018). GFP-PRL-1_G97R and GFP-PRL-1_T13F mutants were generated with the site-directed mutagenesis kit of Agilent (Santa Clara, CA, United States).

JK cells were transfected with the indicated DNA vectors by nucleofection using Amaxa^®^ Cell Line Nucleofector^®^ Kit V and the Amaxa^®^ Nucleofector^®^ II device (Lonza Group). 24 hours after transfection, viable cells were used for experiments after their isolation by centrifugation on Lymphoprep^TM^ (Rafer, Spain).

Genome of JK cells was edited by using the clustered regularly interspaced short palindromic repeats (CRISPR)/Cas9 technology (Mojica and Montoliu, 2016; Torres-Ruiz and Rodriguez-Perales, 2017). Single guides (sg) specific for the exon 2 of *PTP4A1* gene (sgPTP4A1.1: ATGAACCGCCCAGCT CCTGTTG, sgPTP4A1.2: CGAATGCGACCTTAAACAAATT) were cloned using *BsrGI* and *SpeI* restriction sites into the pLVC9 plasmid (Torres-Ruiz and Rodriguez-Perales, 2017), which allows for the expression of the Cas9 endonuclease under the cytomegalovirus (CMV) promoter and of the sgRNA under the U6 promoter. Homologous recombination was promoted by using a donor plasmid (pEGFP-C1, Clontech) containing the GFP sequence and 5′ and 3′ homology arms (HA) (5′HA-GFP-3′HA) (Figure 6A) synthetized by IDT (United States). This plasmid allows for the insertion of the open reading frame of the GFP upstream of, and in-frame with, *PTP4A1* coding sequence and was generated in two steps: initially, a 5′HA-GFP plasmid was constructed by eliminating the promoter region of *GFP* gene in the pEGFP-C1 plasmid (Clontech) with *Ase*I and *Nhe*I digestion and cloning in these restriction sites the 5′HA containing the 685 bp upstream the start codon of genomic *PTP4A1*. Secondly, the 3′HA was introduced into the 5′HA-GFP plasmid using *Xho*I and *Mlu*I restriction sites.

Jurkat cells were transfected using Neon^TM^ transfection system with the parameters: 1.325 V, 10 ms, three pulses. Transfected cells were maintained in culture until the GFP-PRL-1 expression was detected by flow cytometry with a FACSCalibur flow cytometer, (BD Bioscience). Then, edited cells (GFP+) were sorted using a FACSAria III cell sorter (BD Bioscience). PCR of genomic DNA was used to assess the correct edition of the genome and PCR specificity was assessed by nested PCR.

### Immunoprecipitation and Western Blot

For immunoprecipitation, JK cells were lysed for 30 min in ice-cold lysis buffer containing 10 mM Tris–HCl pH 7.5, 0.5% NP-40, 150 mM NaCl, 0.5 mM EDTA, 1× protease inhibitor cocktail, 10 mM NaF, 1 mM PMSF and 1 mM Na_3_VO_4_. Equilibrated GFP-Trap^®^_A beads (ChromoTek, GmbH) were added to the cell lysates and incubated for 2 hours at 4°C with rotation. After two washes, Laemmli buffer was added and beads were boiled for 10 min at 95°C to dissociate complexes. Western Blot was performed with the supernatants.

Boiled samples were separated by SDS-PAGE in 10% acrylamide gels. Proteins were then transferred to an Immobilon-FL transfer membrane. After transference, membranes were blocked with LI-COR blocking buffer (LI-COR Bioscience) before O/N incubation with primary mouse anti-PRL1/2 or rabbit anti-GFP antibodies. Fluorescently labeled secondary antibodies IRDye 680 goat anti-mouse IgG and IRDye 800 goat anti-rabbit IgG (LI-COR Bioscience) were used. Blots were scanned with an Odyssey^®^ Infrared Imager (LI-COR Bioscience).

### Cell-Cell Conjugate Formation, Immunofluorescence and Confocal Microscopy

Jurkat cells were treated for 4 hours with BFA (10 μg/ml) or the vehicle (methanol) and were plated on poly-L-Lysine-coated coverslips for immunofluorescence or Nunc Lab-Tek chambers (Thermo Fisher Scientific) for fluorescence recovery after photobleaching (FRAP) experiments. In order to study cell-cell conjugates, Raji cells were loaded with SEE at 1 μg/mL and labeled with 10 μM CMAC for 1 hour at 37°C. After two washes, Raji cells were mixed with JK cells at a 1:1 cell ratio, briefly centrifuged, and gently resuspended. 50 μL of such mixture was plated on poly-L-Lysine-coated coverslips and cells were allowed to interact for 20 min at 37°C.

Jurkat/Raji cell conjugates or JK cells alone were fixed with 4% paraformaldehyde and permeabilized as indicated with Triton X-100 0.1% by consecutive incubations of 5 min at room temperature (RT). Samples were then blocked with 10 μg/ml human gamma globulin in TNB [20 mM Tris–HCl pH7.5, 150 mM NaCl, 0.5% blocking reagent (Roche Diagnostics, Gmbh)] for 45 min at RT, and stained with the indicated antibodies in TNB for 1 hour at RT. Samples were then washed and incubated with Alexa Fluor 594 conjugated Goat anti-mouse IgG polyclonal antibody at RT for 30 min and mounted in Mowiol on coverslips.

Confocal microscopy was performed with a FV-1200 microscope (Olympus Deutschland GmbH, Germany) and the objective UPLSAPO60XO (NA: 1.35). The excitation lines of 405 nm, 488 nm and 594 nm were used for CMAC, GFP and Alexa Fluor 594, respectively. An excitation filter DM405/488/594 was used and the emitted green and red fluorescence was collected by PMT detectors after sequential passing through an emission filter SDM595 and a mirror (Olympus). The analysis of fluorescence images was done with FIJI freeware (NIH, United States). The ratio of plasma membrane/total GFP-PRL-1 was calculated by using the “Synapse Measures” plugin (Calabia-Linares et al., 2011). Briefly, the average intensity of several ROIs on the cell membrane (<mROIs>), on the total cell (<tROIs>) and on empty areas of the image (for background) was measured. The ratio <mROIs>/<tROIs> was then obtained after background subtraction. The Pearson’s coefficients for colocalization analysis were quantified using the “Colocalization Threshold” plugin.

### Fluorescence Recovery After Photobleaching

Jurkat cells were transfected and treated with BFA or left untreated as indicated before and FRAP experiments were implemented with the FRAP wizard of a confocal microscope FV-1200 equipped with an UPLSAPO60XW objective (NA: 1.20) (Olympus Deutschland GmbH, Hamburg, Germany). Bleaching was done with the 488-laser line at 80% power for 0.2 s. In the post-bleaching step, GFP was excited with a 488-nm laser line at around 1–2 % power and time-lapse frames were acquired every 2 s (2B4-GFP) or 1 s (GFP-PRL-1). An excitation filter DM405/488 was used and the emitted fluorescence was collected by the PMT detector after being reflected by a mirror (Olympus). Digital images were built with a pixel size of around 40 nm. Experimental FRAP data corrected for the bleaching were obtained by:

$$\text{FRAP}=\frac{\left(I_{b}-B_{g}\right)}{\left(I_{c}-B_{g}\right)}$$

Where *I*_*b*_ is the vector of the fluorescence intensity in the bleached area in each frame, *I*_*c*_ the vector of the fluorescence intensity of the cell in each frame and *B*_*g*_ is the vector of the fluorescence intensity of an area without cells (background) in each frame. Vectors of fluorescence intensity (*I*_*b*_, *I*_*c*_, and *B*_*g*_) were obtained with the FIJI freeware (NIH, United States). FRAP curves were then obtained by normalizing experimental FRAP data by its highest value in the *plateau* after bleaching. In order to obtain the half recovery time, normalized FRAP curves where fitted to a model of diffusion in the crowded environment of the cell (Feder et al., 1996) by:

$$F\left(t\right)=\frac{\left[fd+fi{\left(\frac{t}{m}\right)}^{\alpha }\right]}{\left[1+{\left(\frac{t}{m}\right)}^{\alpha }\right]}$$

Where *m* is the half recovery (*t*_1/2_), *fd* is the fluorescence immediately after the bleaching, *fi* is the fluorescence in the *plateau* and α is the anomalous exponent. Data calculation and fitting was implemented in MatLab (MathWorks^®^, United Kingdom).

### Fluorescence Correlation Spectroscopy

Fluorescence correlation spectroscopy experimental data were acquired with a LSM Upgrade Kit (PicoQuant, Berlin, Germany) attached to a FV-1200 microscope equipped with an UPLSAPO60XW objective (NA: 1.20) (Olympus Deutschland GmbH, Hamburg, Germany). For the acquisition of the confocal sections of cells, a 488-laser line was used. An excitation filter DM405/488/559/635 was used and the emitted fluorescence was collected by the PMT detector after being reflected by an emission filter SDM560 (Olympus). Digital images were built with a pixel size of around 40 nm. For FCS measurements, transfected cells with the lowest expression levels were selected in order to obtain good auto-correlation functions (ACFs). The GFP was excited with a 488-nm laser line with the required power to obtain half of the counts per molecule in saturating conditions. The same acquisition filter as before was used and the fluorescence emission was collected by a SPAD detector (Olympus). Experimental autocorrelation curves were obtained from 10 measurements of 10 s per cell and the average curve was used for representation and further analysis. The analysis of the FCS data was performed as described before (Roda-Navarro and Bastiaens, 2014) and experimental curves were fitted to a function with a unique free 3D diffusion component or to a function with two components: one fast with a free 3D diffusion and one slow with a free 2D diffusion:

$$G\left(\tau \right)=\frac{1}{N}\cdot \left[\frac{F_{1}}{\left(1+\frac{\tau }{\tau_{d1}}\right)\cdot \sqrt{1+\frac{\tau }{{\text{s}}^{2}\cdot \tau_{d1}}}}+\frac{1-F_{1}}{\left(1+\left(\frac{\tau }{\tau_{d2}}\right)\right)}\right]$$

Where *N* is the average number of particles in the confocal volume, *F*_1_ is the fraction of the 3D free diffusion component, 1-*F*_1_ is the fraction of the membrane component, τ is the delay time, *s* is the spatial parameter of the focal point and τ_*d*1_ and τ_*d*2_ are the translational diffusion of the 3D and 2D free diffusion components, respectively. Data fitting was implemented in MatLab.

Before FCS measurements in cells, the system was calibrated by measuring the translational diffusion of a 20 nM solution of Atto-488 at 37°C. Knowing the diffusion coefficient of the Atto-488 (400 μm^2^/s), the radius of the confocal point (Wxy) can be calculated with the following equation:

$$D=\frac{W_{xy}^{2}}{4\tau_{d}}$$

Robust measurements of Wxy were obtained along experiments (Wxy = 145 ± 3 nm) and the diffusion coefficients (*D*) of the proteins in the cells were calculated with the same equation by using the τ_*d*1_ and τ_*d*2_ values. The mean ± standard deviation is indicated in the text. The calibration also allows obtaining the *s*, which was then fixed in the diffusion model when experimental ACFs were fitted.

### Statistical Analysis

Statistical analysis was implemented in PRISM 6 (GraphPad Software, San Diego, CA, United States). The used tests and the corresponding *p*-values are indicated in figure legends.

## Results

### GFP-PRL-1 Membrane Accumulation Is Resistant to Brefeldin A Treatment

**FIGURE 1:**
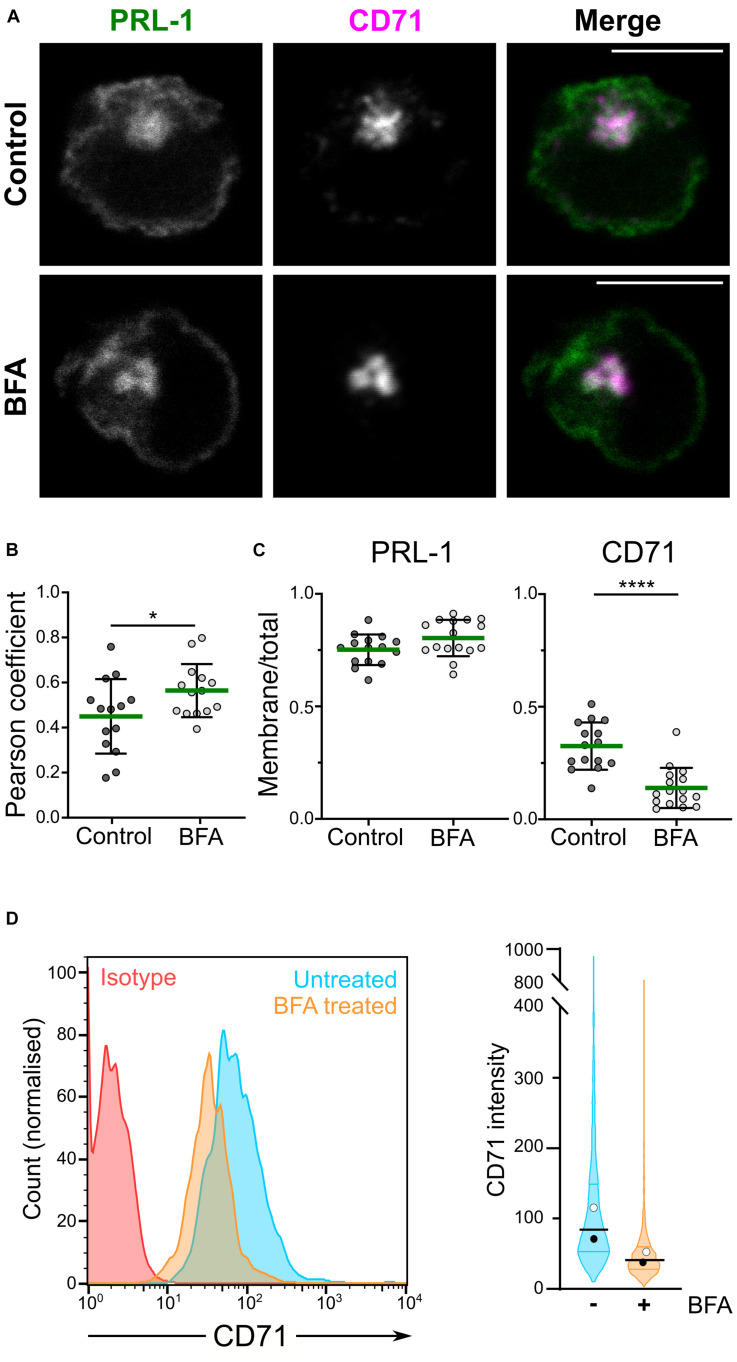
GFP-PRL-1 membrane enrichment is resistant to brefeldin A treatment. **(A–C)** Distribution of GFP-PRL-1 transfected in JK cells untreated (control) or treated with BFA, fixed, permeabilized and stained for CD71. **(A)** Individual channels (gray scale) and merged images (green for GFP-PRL-1 and magenta for CD71) of a confocal section of representative cells are shown. Scale bar: 10 μm. **(B)** Pearson’s correlation coefficients for colocalization of PRL-1 with CD71-containing endosomes. **(C)** Ratio of plasma membrane signal versus total signal of PRL-1 and CD71 before and after BFA treatment. **(B,C)** Dots represent the analyzed cells. Samples were compared by an unpaired Student’s *t* test. *p < 0.05, ****p < 0.0001. Non-significant comparisons are not indicated. **(D)** Left panel: representative histograms of surface expression of CD71 assessed by flow cytometry in samples treated or not treated with BFA for 4 h. Right panel: violin plots depicting the data distribution for all the cells analyzed in two independent experiments (>2000 cells). Samples are color-coded: untreated in cyan and BFA treated in orange color.

Phosphatase of regenerating liver-1 is enriched at the plasma membrane in different cellular models and has been proposed to localize to the ER (Wang et al., 2002), as well as in early endosomes few minutes after endocytosis and in the recycling compartment (Zeng et al., 2000). Recently, we have shown the distribution of PRL-1 and PRL-3 to the CD71-containing recycling compartment in lymphoid cells (Castro-Sanchez et al., 2018; Aguilar-Sopena et al., 2020). These results suggested that, in lymphocytes, the recycling compartment might contribute to a rapid plasma membrane replenishment of endocytosed PRL-1 in the same way it sustains surface expression of CD71 (Klausner et al., 1983). In order to further investigate this hypothesis, we studied the plasma membrane localization of a previously generated GFP-PRL-1 fluorescent fusion protein (Castro-Sanchez et al., 2018) overexpressed in JK cells treated or not with BFA. This compound is a known inhibitor of the conventional secretory pathway (Nickel, 2010) that, by targeting the recycling endosomal compartment in lymphocytes, rapidly blocks the surface expression of the integral membrane proteins CD71 and CD3, which are rapidly recycled to the plasma membrane after endocytosis (Liu et al., 2000). Consistent with a distribution in the recycling compartment, a higher colocalization of PRL-1 with the compacted CD71-containing endosomes was observed in cells treated with BFA (Figures 1A,B). To evaluate the effect of BFA on the accumulation of GFP-PRL-1 at the plasma membrane, we calculated the ratio of plasma membrane versus total protein in treated and untreated cells. While BFA treatment drastically decreased the ratio of membrane CD71, it had no significant effect on GFP-PRL-1 distribution (Figures 1A,C), nor in the total levels of GFP-PRL-1 (Supplementary Figure 1). As expected, a substantial decrease in surface CD71 was consistently observed by flow cytometry (Figure 1D). These data indicated that, by contrast to CD71, plasma membrane PRL-1 does not seem to be replenished by recycling endosomes and suggest a BFA-resistant molecular mechanism to sustain PRL-1 plasma membrane accumulation.

### GFP-PRL-1 Moves Extremely Fast at the Plasma Membrane

**FIGURE 2:**
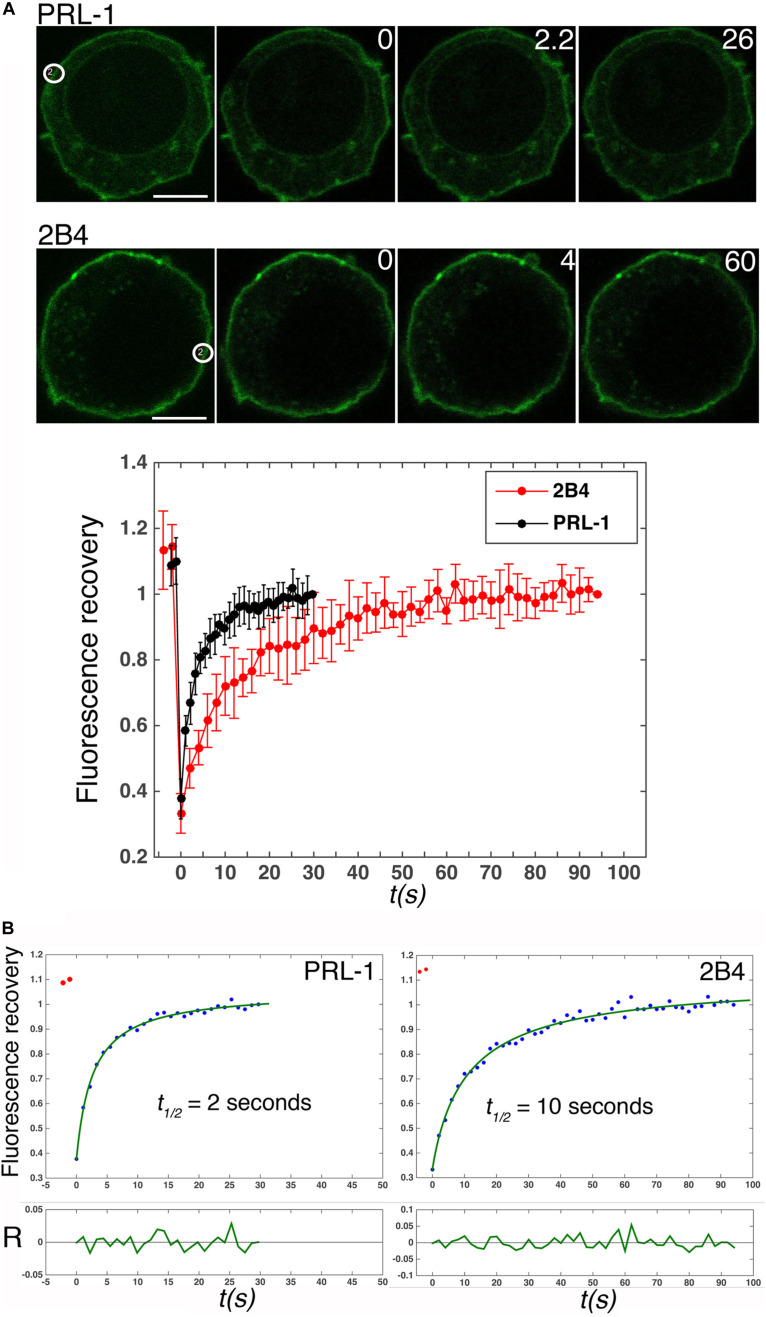
GFP-PRL-1 moves extremely fast at the plasma membrane. **(A)** Upper panels show images of confocal sections of cells transfected with GFP-PRL-1 or 2B4-GFP and studied by FRAP. White circles label the place where bleaching was applied. White numbers in the upper right corner of the images indicate seconds after bleaching. Scale bar: 5 μm. The lower graph represents the average value and standard deviation of FRAP data normalized to the plateau and obtained from *n* = 5 cells (2B4-GFP) or *n* = 9 cells (GFP-PRL-1). *t(s)* indicates time in seconds. **(B)** Fits (green line) of the average values shown in (**A**, lower graph) to the theoretical model described in section “Materials and Methods.” The obtained half fluorescence recovery (*t*_1/2_) is shown. *R* indicates the fit residuals and *t(s)* the time in seconds.

**FIGURE 3:**
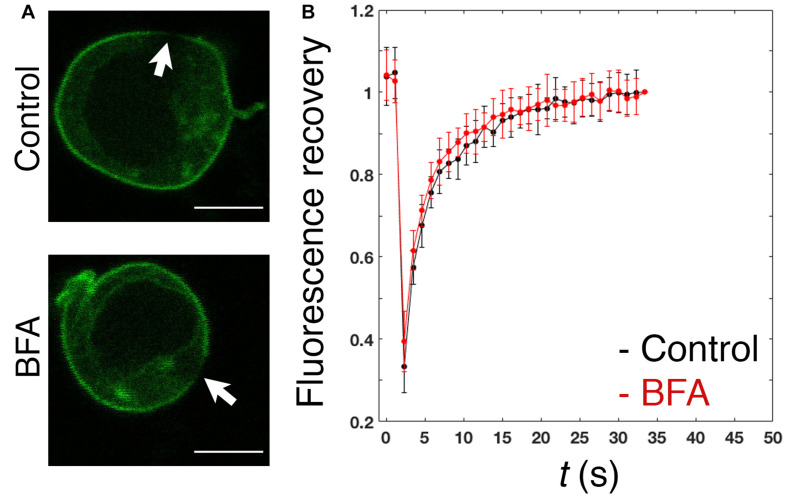
Fast replenishment of PRL-1 at the plasma membrane is resistant to BFA treatment. **(A)** Confocal images of representative cells treated with BFA or the vehicle (control) for 4 h and subsequently measured by FRAP. White arrows label the area where bleaching was applied. Scale bar: 5 μm. **(B)** The graph represents the average value and standard deviation of FRAP data normalized to the plateau and obtained from *n* = 15 control cells or *n* = 16 BFA-treated cells. *t(s)* indicates time in seconds.

In spite of the proposed contribution of the polybasic region to the plasma membrane enrichment (Sun et al., 2005), the dynamics of PRL-1 in live cells has not been studied. Therefore, the mobility of GFP-PRL-1 was assessed in our model by FRAP. GFP-PRL-1 or the GFP-tagged integral membrane receptor 2B4 (2B4-GFP) (Roda-Navarro et al., 2004) were bleached at the plasma membrane and the fluorescence recovery was monitored by time-lapse confocal microscopy. Plasma membrane GFP-PRL-1 showed a five times faster half-recovery time (*t*_1/2_ = 2 s) than the integral membrane receptor 2B4-GFP (*t*_1/2_ = 10 s) and a mobile fraction of around 90% (Figure 2). Consistent with the results obtained by BFA treatment (Figure 1), the half-recovery time of PRL-1 was too rapid to be the result of recycling endosomes targeting the plasma membrane or of vesicular trafficking from the Golgi apparatus in the conventional secretory pathway. Supporting this idea, this fast recovery was resistant to BFA treatment (Figure 3). The half-recovery time and mobile fraction of GFP-PRL-1 was similar to that observed for other substrates of farnesylation or geranylgeranylation proposed to exchange soluble cytosolic and Golgi or ER pools (Goodwin et al., 2005), supporting the notion that GFP-PRL-1 membrane pool might be quickly replenished by a cytosolic diffusing pool of protein. These data showed that GFP-PRL-1 fluorescence recovery at the plasma membrane was mainly mediated by a highly dynamic recycling compartment-independent mechanism, which might be based on lateral diffusion within the membrane, on a rapid exchange of cytosolic and membrane pools of the protein or a combination of both processes.

### Diffusible Cytosolic and Membrane Pools of Overexpressed GFP-PRL-1

**FIGURE 4:**
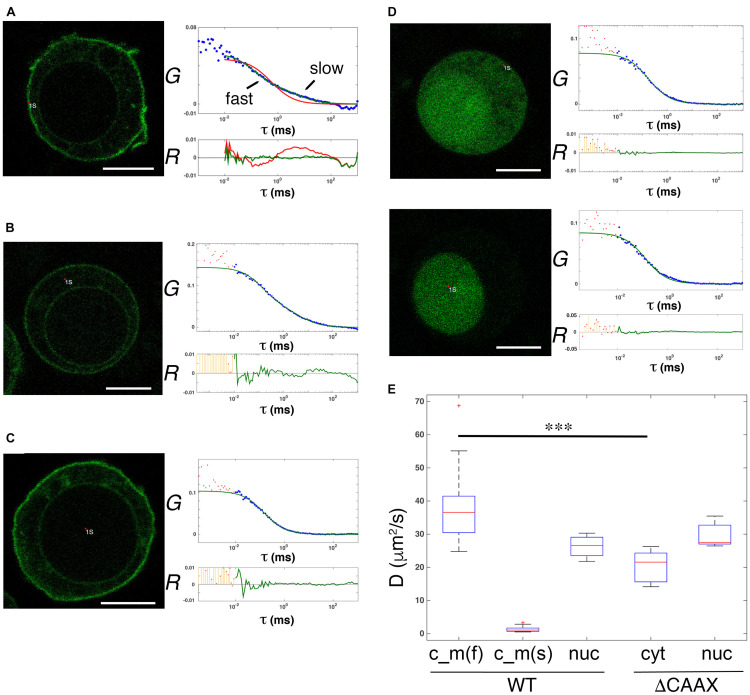
Diffusible cytosolic and membrane pools of overexpressed GFP-PRL-1. **(A–C)** FCS measurements of GFP-PRL-1 wild type obtained in focal points close to the plasma membrane **(A)**, in the internal cytoplasm **(B)** or in the nucleus **(C)**. **(D)** FCS measurements of GFP-PRL-1_ΔCAAX done first at the cortical cytoplasm (upper panels) and secondly in the nucleus (lower panels) of a representative cell. **(A–D)** Representative cells (left images) and experimental ACFs (blue dots) and fits (green or red lines) (upper graphs) and residuals (*R*) of fits (lower graphs) are shown. Scale bar: 5 μm. Experimental ACFs represent the average of 10 measurements. The τ indicates the translational time in milliseconds (ms) and the *G* indicates the autocorrelation. Data above 0.01 ms were fitted with equations shown in section “Materials and Methods.” Experimental data under 0.01 ms are excluded from the fit. **(E)** Boxplot of the calculated diffusion coefficients (*D*). Red line indicates de median, the edges of the box correspond to the 25th and 75th percentiles, the whiskers extend to the most extreme data points and red crosses indicate outliers. Data of GFP-PRL-1 (WT) and GFP-PRL1_ΔCAAX at the different cellular compartments obtained from *n* = 22 cells [WT c_m(f) and c_m(s)], *n* = 4 cells (WT nuc), *n* = 6 cells (ΔCAAX cyt) or *n* = 5 cells (ΔCAAX nuc). c_m indicates measurements done at the membrane **(A)** and the cytoplasm **(B)**. The f and s indicate fast and slow components, respectively. nuc indicates measurements at the nucleus and cyt at the cytosol. The fast diffusion in the different samples was compared by a one-way ANOVA with a Tukey’s multiple comparison test. ^∗∗∗^*p* < 0.001. Non-significant results are not indicated.

To further prove the existence of diffusing soluble and membrane pools of GFP-PRL-1, we evaluated the dynamics of GFP-PRL-1 by FCS. This technique has a higher temporal resolution than FRAP (Kim et al., 2007) and consequently segregates more precisely different pools of fluorescent particles by measuring their diffusion coefficients. Experimental ACFs obtained for GFP-PRL-1 moving through the focal point placed close to the plasma membrane or at the inner cytoplasm in equatorial sections of cells could not be fitted to a theoretical model of three-dimensional (3D) free diffusion. By contrast, ACFs were properly fitted to a theoretical model containing two components: one with a fast 3D free diffusion (<*D*>*_*c_m_*__*fast*_* = 38.7 ± 10.7 μm^2^/s), and one with a slow two-dimensional (2D) free diffusion typical of cell membranes (<*D*>*_*c_m_*__*slow*_* = 1.3 ± 0.7 μm^2^/s) (Figures 4A,B,E). Further supporting the 2D diffusion at the membrane, only the fast component was detected at the center of the nucleus where no membranes were included in measurements (<*D*>*_*wt_nuc*_* = 26.3 ± 3.6 μm^2^/s) (Figures 4C,E). Also, ACFs placed in more apical or basal membranes of the cell (in which probably a higher amount of membrane crosses along the short axes of the focal point) also fitted the 3D–2D model, rendering consistent diffusion coefficients (Supplementary Figure 2). These data indicated the diffusion of GFP-PRL-1 in the cytosol, as well as the plasma and ER membranes. To further prove the existence of a membrane component, ACFs were obtained for a mutant, which lacks the C-terminal CAAX motif for farnesylation, so called GFP-PRL-1_ΔCAAX (Supplementary Figure 3). As previously shown (Zeng et al., 2000; Wang et al., 2002; Castro-Sanchez et al., 2018), the distribution of GFP-PRL-1 to endomembranes and its enrichment at the plasma membrane was strictly dependent on the CAAX motif for farnesylation and the steady-state distribution of this mutant showed a soluble pattern with some accumulation in the nucleus (Figure 4D). ACFs obtained for the mutant in the cortical cytoplasm close to the plasma membrane and in the nucleus of cells were properly fitted to the model with a unique 3D free diffusion component (<*D*>_Δ_*_*CAAX_cyt*_* = 20.6 ± 4.8 μm^2^/s and <*D*>_Δ_*_*CAAX_nuc*_* = 29.7 ± 3.5 μm^2^/s) (Figures 4D,E). These results revealed the existence of two pools of GFP-PRL1, one diffusing in the cytosol and one diffusing in cell membranes. Faster diffusion in the cytosol of GFP-PRL-1 than the GFP-PRL-1_ΔCAAX mutant (Figure 4E) suggested the existence in this compartment of a CAAX motif-mediated mechanism for fast diffusion. Together, FRAP (Figures 2, 3) and FCS data suggest the existence of freely diffusing cytosolic GFP-PRL-1, which might communicate protein pools at endomembranes and the plasma membrane.

### Trimerization Is Not Required for Plasma Membrane Accumulation or Diffusion of GFP-PRL-1

**FIGURE 5:**
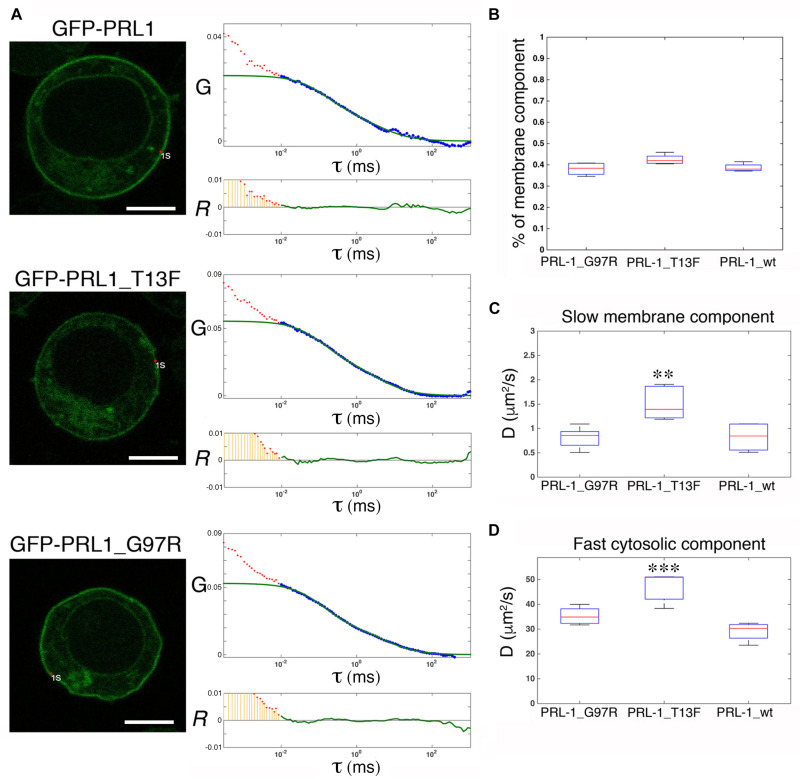
Trimerization is not required for plasma membrane enrichment or diffusion of GFP-PRL-1. **(A)** FCS measurements are shown for either GFP-PRL-1 wild type or the indicated mutants at focal points near the plasma membrane. Representative cells (left images) and graphs with experimental ACFs (dots) and fits (green lines) (upper graphs) and residuals (*R*) of fits (lower graphs) are shown. Scale bar: 5 μm. Experimental ACFs represent the average of 10 measurements. The τ indicates the translational time in milliseconds (ms) and the *G* indicates the autocorrelation. Data above 0.01 ms were fitted with equations shown in section “Materials and Methods.” Experimental data under 0.01 ms are excluded from the fit. **(B)** Boxplot of the percentage of the plasma membrane slow component for each of the studied protein. **(C,D)** Boxplot of the diffusion coefficients (*D*) of the slow **(B)** and the fast **(D)** component for each of the studied protein. **(B,D)** Red line indicates the median, the edges of the box correspond to the 25th and 75th percentiles and the whiskers extend to the most extreme data points. Data were obtained from *n* = 5 (GFP-PRL-1_wt) or *n* = 6 cells (GFP-PRL-1_TF13 and GFP-PRL-1_G97R). Samples were compared by a one-way ANOVA with a Dunnett’s multiple comparison test with the control sample GFP-PRL-1_wt. ^∗∗^*p* < 0.01 and ^∗∗∗^*p* < 0.001. Non-significant results are not indicated.

Structural studies have shown that PRL-1 crystallizes as a trimer, and thus, it has been proposed to represent a mechanism for PRL-1 stabilization at the plasma membrane (Jeong et al., 2005). Therefore, we aimed to further test this idea in live cells by using FCS. It should be noted that following Stokes-Einstein equation, the change in molecular size following trimerization (from around 20 to 60 kDa) is not expected to have a detectable impact on diffusion, and consequently FCS data obtained with GFP-PRL-1 are compatible with the existence of monomers or trimers. Thus, we generated two monomeric mutants, GFP-PRL-1_G97R and GFP-PRL-1_T13F, which change amino acid residues critical for trimerization (Jeong et al., 2005; Sun et al., 2005; Supplementary Figure 3). The steady-state distribution of all the fusion proteins used was similar and the same theoretical model with 3D and 2D freely diffusing components properly fitted the ACFs (Figure 5A). The fraction of the membrane component of the GFP-PRL-1 wild type was not significantly different when compared with GFP-PRL-1_G97R or GFP-PRL-1_T13F mutants (Figure 5B). These data indicated that enrichment at the plasma membrane is not dependent on trimerization. The diffusion coefficients measured for the mutants, in particular for GFP-PRL-1_T13F, were even faster than those measured in these experiments for the GFP-PRL-1 wild type (Figures 5C,D). This result suggests that trimerization would not be required for the diffusion of PRL-1 either in the cytosol or in the plasma membrane.

### Physiological Expression Reveals a Rapid Diffusion of Cytosolic GFP-PRL-1

**FIGURE 6:**
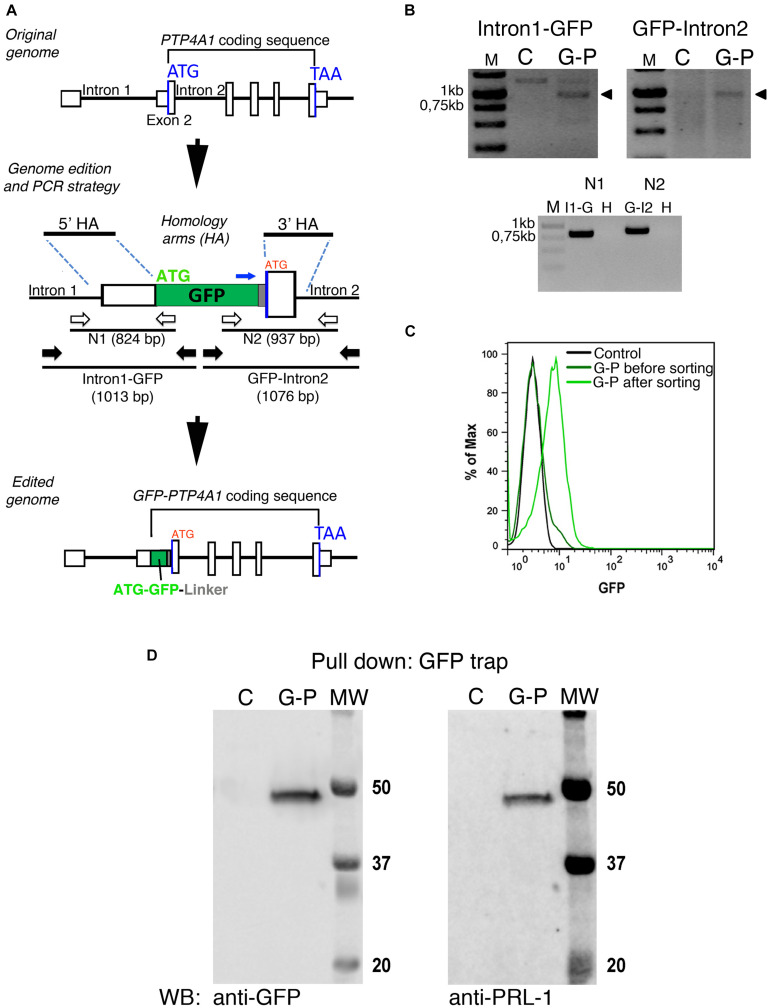
GFP-PRL-1 expression by genome edition of Jurkat cells. **(A)** Upper panel: Schematic representation of the original *PTP4A1* gene. The exons are indicated by white boxes and introns by black lines, the Start (ATG) and End (TAA) codons by blue lines. Middle panel: the plasmid used to insert by homologous recombination the GFP cDNA in-frame with exon 2 coding sequence is shown. The homology arms used for homologous recombination are shown (5′HA and 3′HA). The GFP ATG is labeled in green, while the original ATG of the edited *PTP4A1* gene is labeled in red. PCRs were designed to amplify a DNA fragment covering the *PTP4A1* intron 1 and the 5′ region of the GFP coding sequence (Intron1-GFP) and a fragment covering the 3′ region of GFP coding sequence to *PTP4A1* intron 2 (GFP-Intron2). The primers for these PCRs are shown as black arrows. Additional nested PCRs (N1 and N2) were designed with nested primers (represented by white arrows) to further test specificity of genomic PCRs. PCR products are represented as black lines under primers with their expected product size (bp) indicated. The oligonucleotide used to sequence the joint GFP-linker-PRL-1 is indicated with a blue arrow. Lower panel: Schematic representation of the edited *GFP-PTP4A1* gene. **(B)** PCRs indicated in panel **(A)** were run in agarose gels and visualized with ethidium bromide staining. Upper panels: Intron1-GFP and GFP-Intron2 PCRs of genomic DNA of edited (G-P) or unedited control (C) cells. Arrowheads indicate the PCR products of the expected size. Lower panel: nested N1 and N2 PCRs of Intron1-GFP (I1-G) and GFP-Intron2 (G-I2) or water (H) as negative control. Size markers (M) are indicated in kilo base pairs (Kb). **(C)** Normalized histogram of the GFP fluorescence of control and edited JK (G-P) cells before and after sorting. **(D)** Immunoprecipitation of GFP-PRL-1 followed by WB with the indicated antibodies. Numbers indicate the weight (kDa) of each molecular weight marker (MW), C indicates control unedited cells and G-P the edited cells.

**FIGURE 7:**
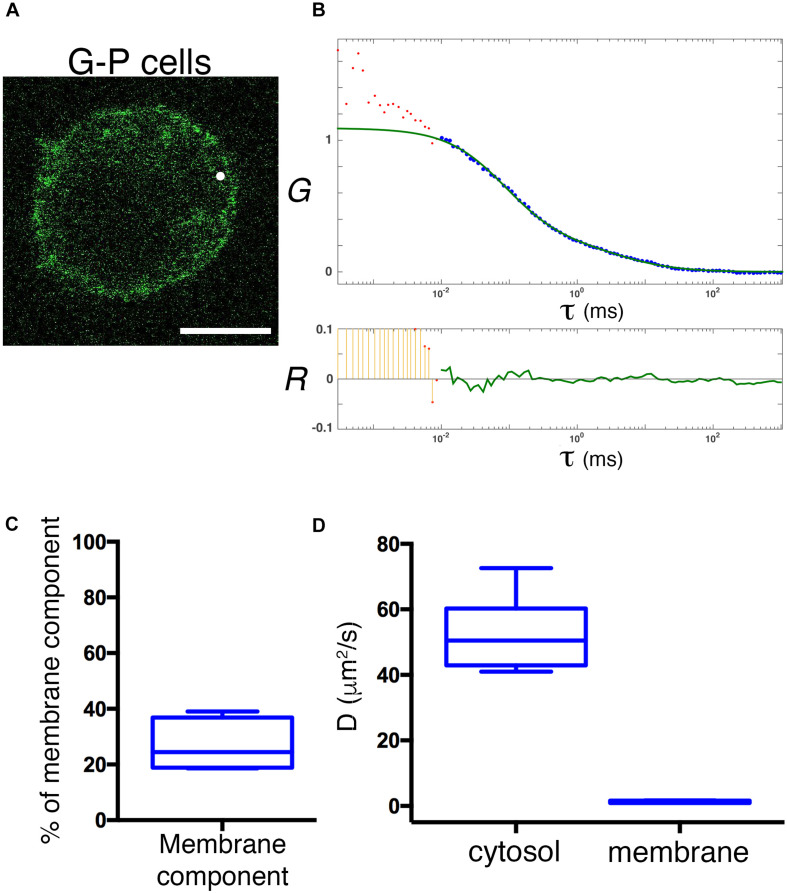
Physiological expression reveals a rapid diffusion of cytosolic GFP-PRL-1. **(A)** Confocal section of a representative G-P cell shows the steady-state distribution of GFP-PRL-1. The white spot indicates the position of the focal point for the FCS measurements. Scale bar: 5 μm. **(B)** Experimental ACF (dots) of GFP-PRL-1 obtained in the cell shown in panel **(A)**. Data represent the average of 10 measurements. The fit (green line) and the residuals of the fit (lower panel) are shown. Data above 0.01 ms were fitted with equations shown in material and methods. Experimental data under 0.01 ms are excluded from the fit. The *G* indicates the autocorrelation and the τ the translational time in milliseconds (ms). **(C)** Box plot representation of the membrane component obtained from measurements done in *n* = 6 cells. **(D)** Box plot representation of the diffusion coefficients (*D*) of the fast and slow components obtained in measurements done in *n* = 6 cells. **(C,D)** Middle line indicates de median, the edges of the box correspond to the 25th and 75th percentiles and the whiskers extend to the most extreme data points.

Ectopic overexpression of GFP fluorescent fusion proteins may perturb protein dynamics, the stoichiometry of molecular complexes and functional outputs (Doyon et al., 2011; Gibson et al., 2013). Thus, in order to achieve physiological expression levels of GFP-PRL-1, we established a protocol to edit the genome of JK cells by using CRISPR/Cas9 methodology (Ran et al., 2013; Mojica and Montoliu, 2016; Torres-Ruiz and Rodriguez-Perales, 2017). The GFP cDNA was inserted upstream and in-frame with the *PTP4A1* gene, and the genome edition was verified by PCR (Figures 6A,B) and Sanger sequencing (Supplementary Figure 4). The initial population of GFP positive cells was sorted in order to have cells with homogeneous levels of fluorescence that were called G-P cells (Figure 6C). Then, expression of endogenous GFP-PRL-1 without any detectable free GFP was corroborated by immunoprecipitation and western blot (Figure 6D). Consistently with the literature (Wang et al., 2002), endogenous GFP-PRL-1 in G-P cells redistributed to the mitotic spindle in the dividing G-P cells present in analyzed samples (Supplementary Figure 5A). Further, when G-P cells were activated by antigen presenting cells, they assembled an immunological synapse (IS) in which GFP-PRL-1 and CD3 were accumulated as previously described (Castro-Sanchez et al., 2018; Supplementary Figure 5B). Having seen the correct behavior of GFP-PRL-1, we studied the diffusion of GFP-PRL-1 in G-P cells. Although the steady-state distribution was similar in G-P cells and JK cells overexpressing the protein, with notable accumulation of the GFP-PRL-1 at the plasma membrane, the location at the perinuclear ER was less clear in G-P cells (Figure 7A). Consistently, when FCS data were fitted to the theoretical 3D–2D model detailed in materials and methods, the percentage of the membrane component of ACFs was lower (<*F*>*_*slow*_* = 27 ± 9.2%) than the one obtained with ectopic expressed protein (Figures 7B,C). This indicated that ectopic expression overestimates the fraction of PRL-1 diffusing at membranes. The diffusion coefficient of the fast component of GFP-PRL-1 expressed in G-P cells (<*D*>*_*fast*_* = 52.4 ± 11.7 μm^2^/s) appears to be slightly faster than the one obtained in cells overexpressing the protein, while the diffusion of the slow component (<*D*>*_*slow*_* = 1.2 ± 0.3 μm^2^/s) was similar (Figure 7D). Similar results were obtained when ACFs were fitted with a diffusion model containing a component with 3D diffusion as before and a component with 2D diffusion in a Gaussian elliptical detection volume (Ries and Schwille, 2006; Supplementary Figure 6). These parameters showed that although ectopic overexpression did not qualitatively affect the steady-state distribution of the protein, it did overestimate the membrane fraction of GFP-PRL-1 and mask the fast diffusion of the cytosolic pool of the protein.

## Discussion

Enrichment of CAAX motif-containing proteins in the plasma membrane has been shown to be dependent on second signals, including polybasic regions and palmitoylation (Choy et al., 1999; Resh, 2006). However, the mechanism is often not known. Here, we show that in lymphoid cells the plasma membrane enrichment of PRL-1 is independent of the recycling endosomal compartment or of stabilization by protein trimerization. Instead, there is a soluble cytosolic protein pool, which might be available for a fast diffusion-mediated transport to the plasma membrane.

Brefeldin A treatment disorganizes the traffic between the ER and the Golgi apparatus and in the case of lymphocytes hampers the plasma membrane expression of proteins trafficking in the recycling compartment, including CD3 and CD71 (Liu et al., 2000). Despite being located at early endosomes immediately formed after endocytosis and at the CD71 containing recycling endosomes (Zeng et al., 2000; Castro-Sanchez et al., 2018), BFA treatment shows that the recycling compartment is not involved in a rapid replenishment of plasma membrane PRL-1. The resistance to BFA treatment has also been reported for plasma membrane enrichment of K-Ras, which also contains a polybasic region and a CAAX motif for farnesylation, but not for plasma membrane enrichment of H-Ras and N-Ras, which contain palmitoylation sites instead of polybasic regions (Apolloni et al., 2000). Thus, it seems that plasma membrane accumulation of polybasic/CAAX-containing proteins is mediated by a transport not dependent on the BFA-sensitive secretory pathway or the recycling compartment. By contrast, palmitoylation directs prenylated proteins from the ER to the Golgi apparatus and the secretory pathway, like in the case of H-Ras and N-Ras (Apolloni et al., 2000; Resh, 2006). The binding of PRL-1 to phosphoinositides (Sun et al., 2007; McParland et al., 2011) might constitute a mechanism for selective partitioning to the endosomal compartment and the plasma membrane. However, it should also be noted that the CAAX motif of PRLs contains a second cysteine residue at the amino acid position 171 (CC_171_AX), which has been proposed to be palmitoylated *in vitro*, at least in PRL-3 (Nishimura and Linder, 2013). This would suggest the existence of different post-translational processing fates and this idea deserves further research.

Fluorescence recovery after photobleaching experiments show a very fast and almost complete BFA-resistant recovery of GFP-PRL-1 in bleached membranes. This result suggests a recycling compartment-independent replenishment of plasma membrane PRL-1 and is consistent with both lateral diffusion within membranes and a rapid exchange of PRL-1 with the cytosolic fast diffusing component measured by FCS. The time resolution of our FRAP experiments does not allow the measurement of this fast diffusing cytosolic component. However, farnesylated proteins with similar half-recovery times in FRAP experiments have been proposed to rapidly exchange diffusing pools of cytosolic and Golgi or ER protein (Goodwin et al., 2005). Indeed, FCS data show the existence of freely diffusing cytosolic and membrane pools of GFP-PRL-1 and the calculated diffusion coefficients of these pools are also similar to those obtained for farnesylated proteins (Goodwin et al., 2005). Therefore, it is possible a model in which the PRLs can efficiently but reversibly bind endomembranes and are trapped at the acidic inner leaflet of the plasma membrane by the polybasic amino acid residues. This would work in a similar way to the previously proposed “kinetic trap” model based on palmitoylation of farnesylated proteins (Shahinian and Silvius, 1995). Nonetheless, trapping by the interaction of polybasic residues and acid lipids might be weaker than membrane insertion through palmitoyl groups, as previously discussed (Resh, 2006), rendering the fast dynamics observed in our work.

The position of the CAAX motifs found in the crystal of PRL-1 (facing one side of the trimer) would suggest that trimerization might assist in membrane protein stabilization (Sun et al., 2005). Nonetheless, monomeric mutants did not show a decreased membrane fraction compared to the wild type. Thus, trimerization does not seem to represent a required mechanism for stabilization of PRL-1 at the plasma membrane. Interestingly, the theoretical model of diffusion used here shows a cytosolic component of 3D free diffusion of GFP-PRL-1, which represents around 73% of the tracked protein. A similar result was seen when data were fitted to a model combining a component with 3D free diffusion with a component with 2D diffusion in a Gaussian elliptical detection volume that conceptually fit to the equatorial location of the focal point used in our experiments. The ACFs obtained do not support a direct transport and consequently indicate that PRL-1 is not actively transported on cytoskeleton fibers. Cytosolic free diffusion poses the question about the mechanism mediating its solubilisation. *In vitro* farnesylated proteins have a weak affinity for membranes (Silvius and l’Heureux, 1994) and this would indicate that PRL-1 does not need any solubilising factor, being easily exchanged between the cytosol and cell membranes. It might be possible that trimerization assists in the solubilisation of the protein in the cytosol, perhaps by generating a “farnesylation switch,” in which the farnesyl group would be sequestered by the protein itself. Our data, however, do not support this idea, as monomeric mutants show even faster diffusion in the cytosol, in particular the T13F mutant. These results indicate that diffusion of PRL-1 monomers in the cytosol might be assisted by solubilising factors of farnesylated proteins, such as phosphodiesterase 6-delta subunit (PDEδ), which enables proper solubilisation of other farnesylated proteins, such as K-Ras (Chandra et al., 2011; Schmick et al., 2014). However, overexpressed GFP-PRL-1 shows a faster diffusion in the cytosol than the GFP-PRL-1_ΔCAAX mutant, which would be unable to bind solubilising factors and consequently would be smaller and would show faster diffusion. Supporting the existence of a mechanism generating fast diffusion of PRL-1 in the cytosol, the diffusion of the GFP-PRL-1 expressed at physiological levels in genome-edited G-P cells (52.4 ± 11.7 μm^2^/s) tends to be faster than the overexpressed in transfected cells (38.7 ± 10.7 μm^2^/s) or than the GFP overexpressed alone in our JK cell line or in other systems (25–30 μm^2^/s) (Dayel et al., 1999). This might be due to an excess of overexpressed GFP-PRL-1, which would not be able to bind solubilising factors for proper solubilisation and diffusion. Nonetheless, crowding effects or abnormal structural features generated by the overexpression cannot be ruled out. Previously, it has been shown that overexpression has a dramatic impact on molecular dynamics (Doyon et al., 2011). Thus, our study supports the notion that physiological expression of fluorescent fusion proteins by genome edition will be necessary to obtain more reliable quantitative measurements of molecular dynamics and biochemical reactions in living cells, as recently proposed (Eckert et al., 2020).

**FIGURE 8:**
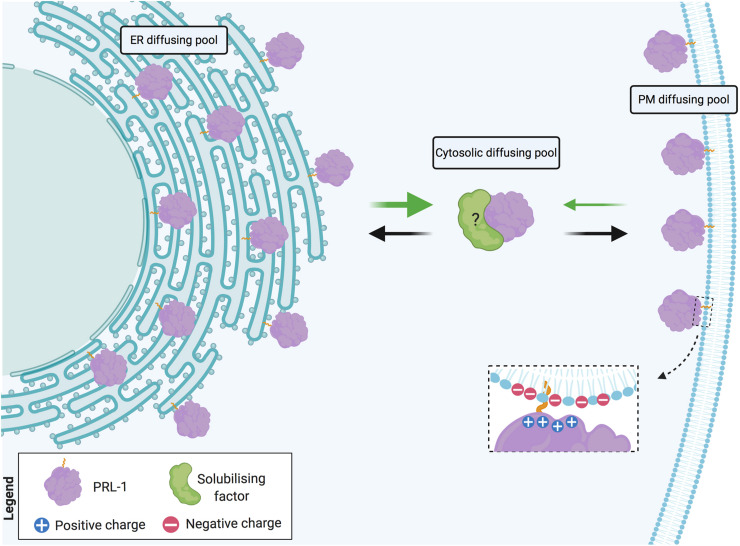
Model of PRL-1 plasma membrane and ER exchange by fast diffusion in cytosolic gaps. PRL-1 is represented by the purple element. Dashed inset details potential interaction, where the plus symbols represent the polybasic sequence and the minus symbols, the acidic residues at the plasma membrane. Black and green arrows indicate the herein proposed PRL-1 exchange between the plasma membrane (PM) and the endoplasmic reticulum (ER). The thicker green arrow represents an easier detachment from the ER membrane due to the absence of acidic residues. The possible existence of a solubilizing factor for PRL-1 is indicated by a green element with a question mark. Created with BioRender.com.

Altogether, these data support the notion of a fast and reversible exchange between cytosolic and membrane pools of PRL-1. Being a farnesylated molecule, intracellular PRL-1 extensively locates at the ER membranes (Wang et al., 2002). In cells with highly packaged ER, such as lymphocytes, a direct transport of proteins from the cortical ER to the plasma membrane has been proposed. This sort of transport might be assisted by direct SNARE-mediated fusion of ER-derived vesicles (Gagnon et al., 2002) or might directly occur through small cytosolic gaps between cortical ER and the plasma membrane where, for example, exchange of lipids takes place (Baumann et al., 2005). PRL-1 readily diffuses in the ER membrane and, consequently, might be rapidly transported from the perinuclear to the cortical ER, from where the low affinity of the binding might enable the release of the protein facilitating the targeting to acidic residues in the proximal plasma membrane (Figure 8). Pathways for rapid and direct transport of proteins from the cortical ER to the plasma membrane are further discussed elsewhere (Nickel, 2010).

All determinants of intracellular traffic, including farnesylation and the polybasic region, seem to be important for plasma membrane and endosomal localization, as well as for the role of PRLs in cell growth and migration or cancer progression (Sun et al., 2007; Campbell and Zhang, 2014; Rubio and Kohn, 2016; Hardy et al., 2018). It is possible that PRL-1 (and other PRLs) traffics to the endosomal compartment to regulate endocytosis or the endosomal fusion with the plasma membrane during exocytosis. Perhaps this function might be accomplished through actin cytoskeleton regulation, which has been previously proposed for PRLs by different authors (Fiordalisi et al., 2006; Nakashima and Lazo, 2010; Gari et al., 2016; Zhan et al., 2016; Castro-Sanchez et al., 2018). At any event, cytosolic diffusion might mediate a rapid coordination of the function of PRLs in different compartments of the cell, such as local areas of the plasma membrane or the mitotic spindle (Wang et al., 2002; Castro-Sanchez et al., 2018). Thus, the dynamics of PRLs might be essential for the cell responses in health and disease, and should be further investigated.

## Data Availability Statement

The raw data supporting the conclusions of this article will be made available by the authors, without undue reservation.

## Author Contributions

All authors contributed to the experimental work and reviewed the manuscript. RT-R and SR-P designed the strategy for the CRISPR/Cas9 genome edition. PR-N conceived the research and wrote the manuscript.

## Conflict of Interest

The authors declare that the research was conducted in the absence of any commercial or financial relationships that could be construed as a potential conflict of interest.
