# Supplementary material for: Fast Diffusion Sustains Plasma Membrane Accumulation of Phosphatase of Regenerating Liver-1
Source: Front Cell Dev Biol. 2020 Dec 4;8:585842. doi: 10.3389/fcell.2020.585842 (PMC7793866; doi:10.3389/fcell.2020.585842)
Supplement: Supplementary file 1 [file Data_Sheet_1.PDF]

Supplementary Material

Fast diffusion sustains plasma membrane accumulation of phosphatase of regenerating liver-1

Castro-Sánchez P[1], Hernández-Pérez S[1], Aguilar-Sopeña O[1], Ramírez-Muñoz R[1], Rodríguez-Perales S[2]*, Torres-Ruiz R[2]* and Roda-Navarro P[1]*

[1]Department of Immunology, Ophthalmology and ENT. School of Medicine, Universidad Complutense de Madrid and 12 de Octubre Health Research Institute (imas12), Madrid, Spain.

[2]Molecular Cytogenetics and Genome Editing Unit, Human Cancer Genetics Program, Centro Nacional de Investigaciones Oncológicas (CNIO), Madrid, Spain.

* Corresponding authors

1 Supplementary Figures and Tables

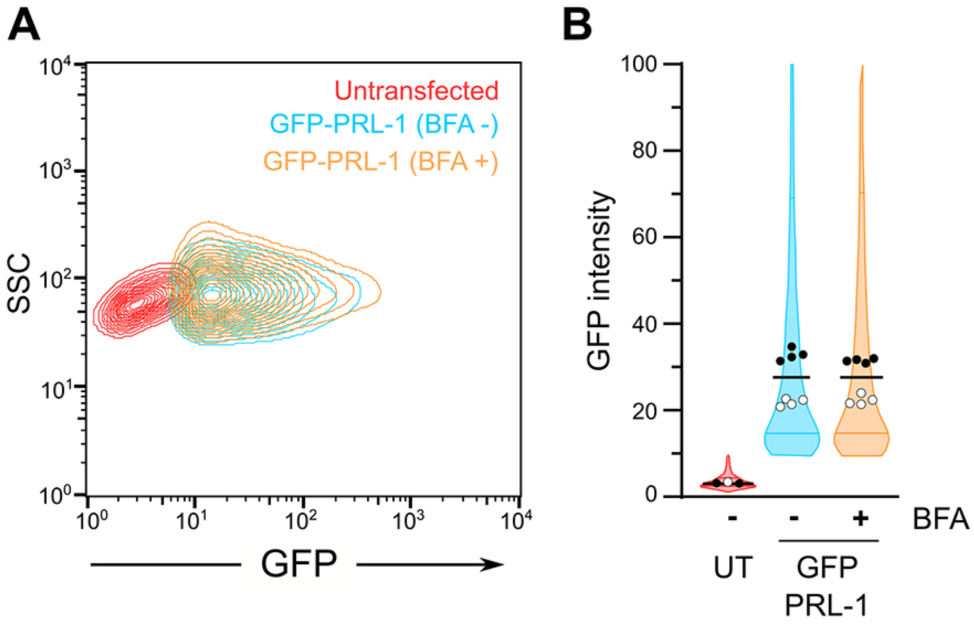

Supplementary Figure 1: Expression levels of transiently transfected GFP-PRL-1 in JK cells before and after BFA treatment. (**A**) Contour plot of the cell complexity (SSC) as a function of the GFP fluorescence intensity of transfected or non-transfected cells. Samples and treatment of each population are indicated. (**B**) Violin plots representing the data of two independent experiments performed as in figure 1. Dots represent the GFP intensity mean values of measurements done in each experiment (4 duplicates per experiment; different experiments coded by black and white colors). UT: untransfected cells. (**A** and **B**) Samples and treatments are color coded: untransfected in red, transfected and untreated in cyan and transfected and BFA-treated in orange.

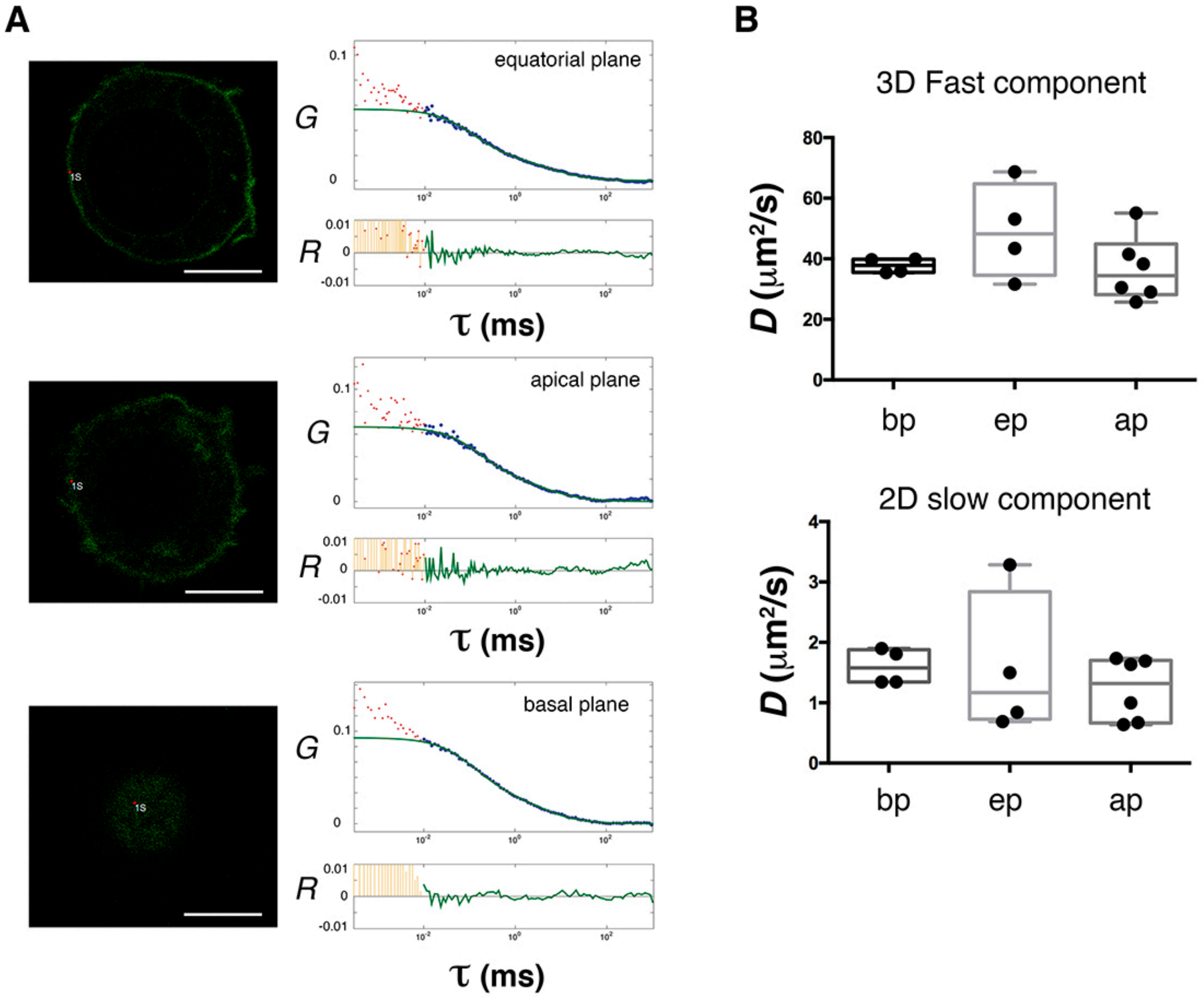

Supplementary Figure 2: FCS measurements in different focal planes. (**A**) Left panels show representative measured cells. Scale bar =5 μm. The "1s" annotation indicates the position of the focal point. Right panels show the ACFs. The blue and red dots indicate the fitted and fitted-excluded experimental data, respectively. The green line represents the fit and the residuals *R* are represented in the lower graph. The *G* and the τ indicate the autocorrelation and the translational time in milliseconds (ms), respectively. (**B**) Box plot of the diffusion coefficients (*D*) obtained for the fast and the slow components. Black dots indicate the cells measured. The bp (basal plane), ep (equatorial plane) and ap (apical plane) samples were compared with a one-way ANOVA with a Tukey's multiple comparison test. Non-significant differences were obtained.

A B

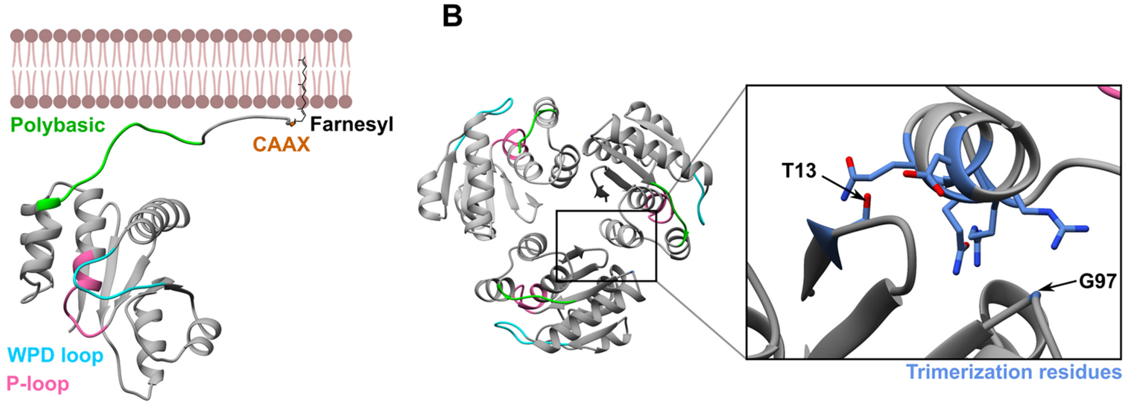

C

```
PRL-1_WT        MARMNRPAPVEVTYKNMRFLITHNPTNATLNKFIEELKKYGVTTIVRVCEATYDTTLVEK   60
PRL-1_ΔCAAX     MARMNRPAPVEVTYKNMRFLITHNPTNATLNKFIEELKKYGVTTIVRVCEATYDTTLVEK   60
PRL-1_G97R      MARMNRPAPVEVTYKNMRFLITHNPTNATLNKFIEELKKYGVTTIVRVCEATYDTTLVEK   60
PRL-1_T13F      MARMNRPAPVEVFYKNMRFLITHNPTNATLNKFIEELKKYGVTTIVRVCEATYDTTLVEK   60
                            *

PRL-1_WT        EGIHVLDWPFDDGAPPSNQIVDDWLSLVKIKFREEPGCCIAVHCVAGLGRAPVLVALALI   120
PRL-1_ΔCAAX     EGIHVLDWPFDDGAPPSNQIVDDWLSLVKIKFREEPGCCIAVHCVAGLGRAPVLVALALI   120
PRL-1_G97R      EGIHVLDWPFDDGAPPSNQIVDDWLSLVKIKFREEPRCCIAVHCVAGLGRAPVLVALALI   120
PRL-1_T13F      EGIHVLDWPFDDGAPPSNQIVDDWLSLVKIKFREEPGCCIAVHCVAGLGRAPVLVALALI   120
                                                    *

PRL-1_WT        EGGMKYEDAVQFIRQKRRGAFNSKQLLYLEKYRPKMRLRFKDSNGHRNNCCIQ          173
PRL-1_ΔCAAX     EGGMKYEDAVQFIRQKRRGAFNSKQLLYLEKYRPKMRLRFKDSNGHRNN----          169
PRL-1_G97R      EGGMKYEDAVQFIRQKRRGAFNSKQLLYLEKYRPKMRLRFKDSNGHRNNCCIQ          173
PRL-1_T13F      EGGMKYEDAVQFIRQKRRGAFNSKQLLYLEKYRPKMRLRFKDSNGHRNNCCIQ          173
                                                                 ****
```

Supplementary Figure 3: PRL-1 structure. (**A**) Schematic view of PRL-1 bound to the plasma membrane. The C-terminal region of PRL-1 contains a polybasic sequence (green) and a farnesylated CAAX sequence (orange), which mediates membrane insertion. The functional domains of the protein (WPD loop in cyan, and P-loop in magenta) remain exposed after membrane insertion. Protein structure has been obtained from the Protein Data Bank (PDB ID 1XM2). Amino acids 157-173 have been added with UCSF Chimera (http://www.rbvi.ucsf.edu/chimera) using a random conformation for representation purposes. (**B**) Schematic view of a PRL-1 trimer (PBD ID 1XM2). Residues involved in trimerization are shown in the inset in blue. Residues T13 and G97 (marked with arrows) were mutated in the present study to prevent PRL-1 trimerization. (**C**) The amino acid sequences of the wild type (WT) protein and mutants used in this work (PRL-1_ΔCAAX, PRL-1_G97R and PRL-1_T13F) have been aligned using UniProt. An asterisk (*) indicates positions of mutated/deleted amino acid residues in PRL-1_G97R, PRL-1_T13F and PRL-1_ΔCAAX, which lacks the C-terminal CAAX motive to prevent membrane anchoring. Different domains of the protein have been colored for better understanding, matching those colors shown in the 3D protein structure in (A) and (B).

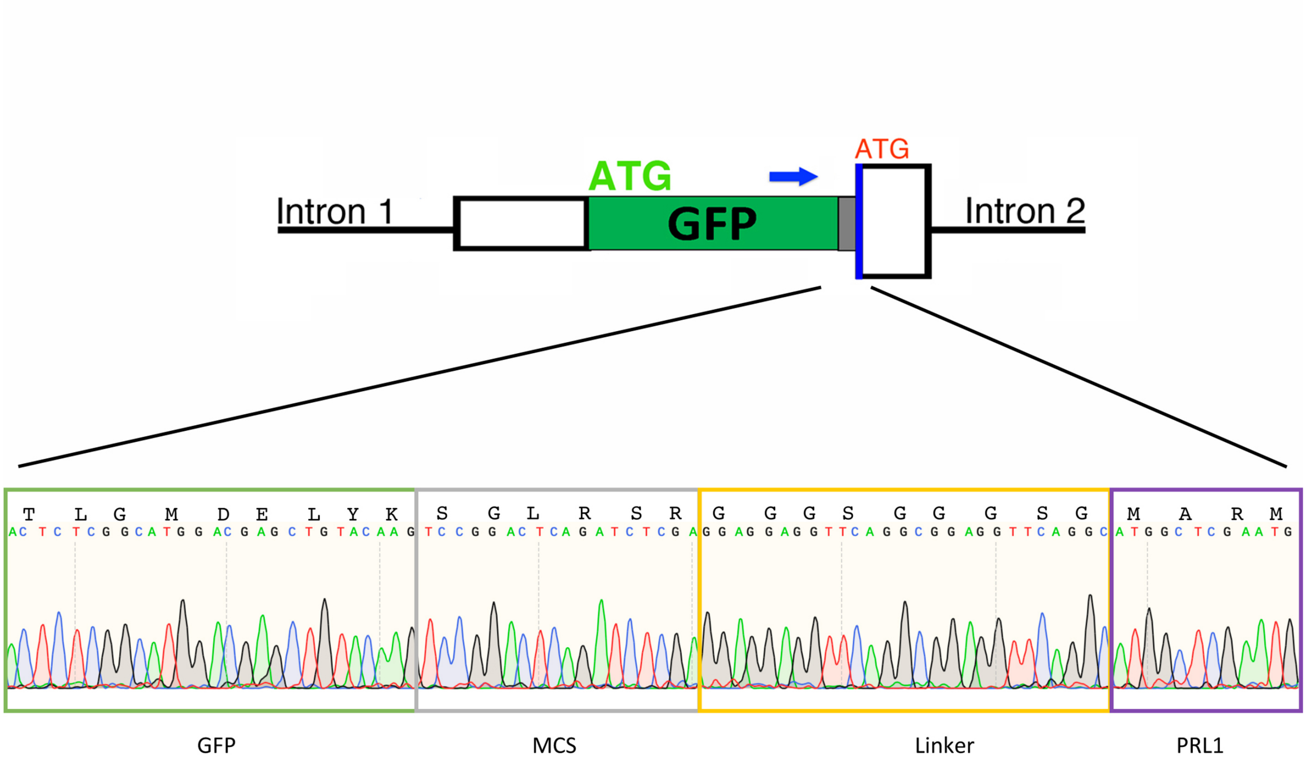

Supplementary Figure 4: Sequence of the GFP-MCS-Linker-PRL-1 site of edited *PTP4A1* gene. Upper schematic represents the position of the GFP regarding the ATG of the *PTP4A1* gene. The Grey box represents the joining sequence composed by a fragment of the multicloning site (MCS) of the Clontech plasmid and the linker inserted. In the lower panel, it is shown the chromatogram of the reading frame expanding the end of the GFP (green box), the MCS (grey box), the linker (yellow box) and the initial nucleotides of PRL-1 (purple box) obtained by sequencing the N2 nested PCR with the oligonucleotide indicated in figure 5.

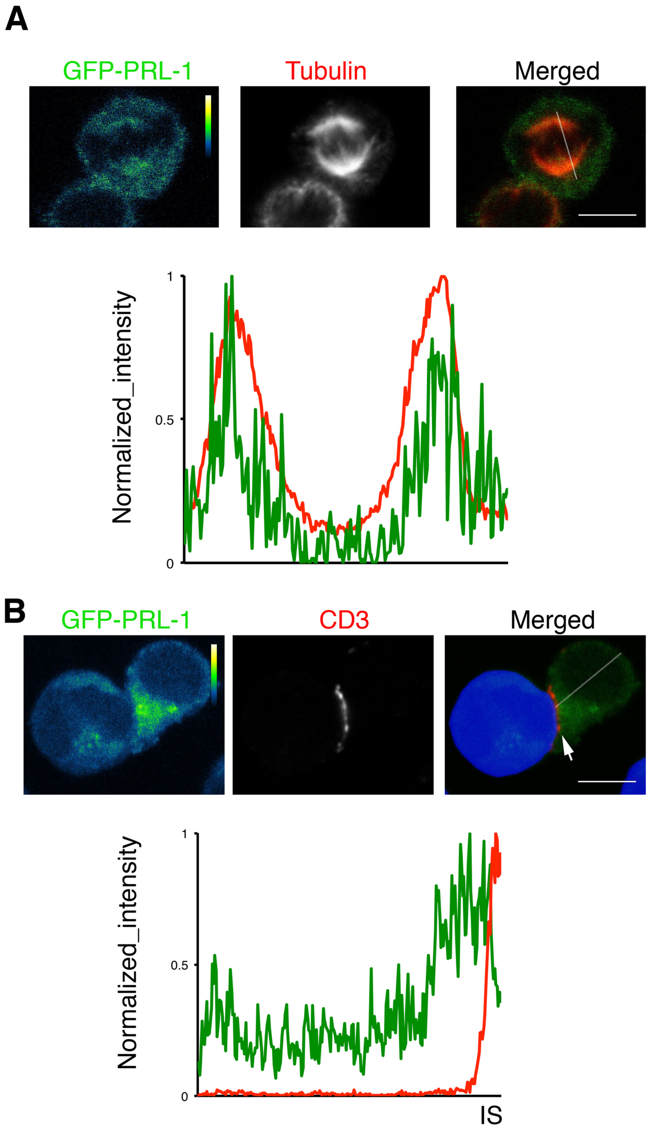

Supplementary Figure 5: Distribution of GFP-PRL-1 expressed under physiological levels. (A) A representative mitotic G-P cell expressing endogenously GFP-PRL-1 in fixed and permeabilized specimens, as indicated in material and methods, is shown in the upper panels. The green (pseudo-color, GFP-PRL-1) and red (grey scale, tubulin) channels and the image of merged channels are shown. Scale bar = 10 µm. Calibration bar for pseudo-color is shown. The normalized intensity profile of the green and the red channels obtained along the white line drawn on the merged image is shown in the lower graph. (**B**) A representative IS established by a G-P cell interacting with a SEE-loaded and CMAC labeled (blue) antigen presenting cell is shown in the upper panels. Scale bars = 10 µm. The green (pseudo-color, GFP-PRL-1) and red (grey scale, CD3) channels and the image of merged channels are shown. Calibration bar for pseudo-color is shown. A white arrow in the merged image points to the IS. The lower panel shows the normalized intensity profile of the green and the red channels obtained along the white line drawn on the merged image. The position of the IS in the profile is indicated.

3D-2D model

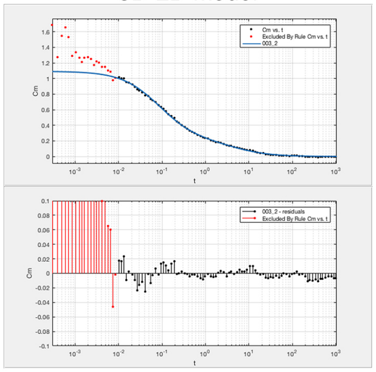

Coefficients (with 95% confidence bounds):
N = 0.9155 (0.9055, 0.9254)
d1 = 0.08814 (0.08247, 0.09381)
d2 = 4.773 (4.016, 5.53)
f1 = 0.8035 (0.7911, 0.8159)

Goodness of fit:
SSE: 0.005688
R-square: 0.9994
Adjusted R-square: 0.9993
RMSE: 0.00843

3D-GE model

$$G(\tau) = \frac{1}{N} \cdot \left[\frac{F_1}{\left(1+\frac{\tau}{\tau_{d1}}\right) \cdot \sqrt{1+\frac{\tau}{s^2.\tau_{d1}}}} + (1-F_1)\left(1+\frac{\tau}{\tau_{d2}}\right)^{-\frac{1}{2}} . \left(1+\frac{\tau}{s^2\tau_{d2}}\right)^{-\frac{1}{2}} \right]$$

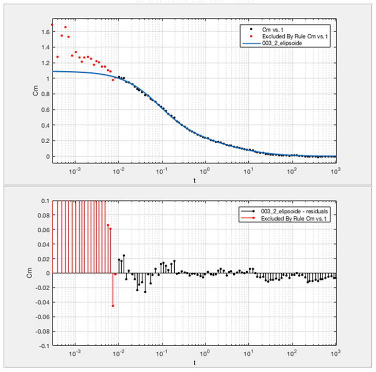

Coefficients (with 95% confidence bounds):
N = 0.9167 (0.9056, 0.9278)
d1 = 0.0887 (0.08075, 0.09665)
d2 = 2.289 (0.7461, 3.833)
f1 = 0.8016 (0.777, 0.8263)

Goodness of fit:
SSE: 0.006104
R-square: 0.9993
Adjusted R-square: 0.9993
RMSE: 0.008735

Supplementary Figure 6: FCS data fit a 3D-2D-Gausian ellipsoid model of diffusion. Comparison of the results obtained when FCS data were fitted to the 3D-2D model of diffusion detailed in material and methods (upper panel) and the shown model, which contains a component with a 3D diffusion and a component with a 2D diffusion in a Gaussian elliptical (GE) detection volume (lower panel). Shown data are obtained from measurements done with G-P cells. The obtained parameters and the goodness of fit are shown.
